# Evaluation of the Cell Population of the Seminiferous Epithelium and Spermatic Indexes of the Bat *Sturnira lilium* (Chiroptera: Phyllostomidae)

**DOI:** 10.1371/journal.pone.0101759

**Published:** 2014-07-08

**Authors:** Danielle B. Morais, Mirlaine S. Barros, Tarcízio A. R. Paula, Mariella B. D. Freitas, Marcos L. M. Gomes, Sérgio L. P. Matta

**Affiliations:** 1 Department of Morphology, Federal University of Rio Grande do Norte, Natal, Rio Grande do Norte, Brazil; 2 Department of Animal Biology, Federal University of Viçosa, Viçosa, Minas Gerais, Brazil; 3 Department of Veterinary, Federal University of Viçosa, Viçosa, Minas Gerais, Brazil; 4 Department of General Biology, Federal University of Viçosa, Viçosa, Minas Gerais, Brazil; University Hospital of Münster, Germany

## Abstract

Due to the scarcity of information about patterns of spermatogenesis in bats, this study aimed to provide information on the testicular activity of the bat *Sturnira lilium* along the annual seasons. Thus, a series of morphometrical and stereological analyses were made using the testes of adult *S. lilium* in order to achieve a better understanding of the sperm production dynamics. Light and transmission electron microscopy analyses were performed in testicular fragments of animals captured during dry and rainy seasons. The testes followed the pattern of organization described for other mammals, and there were no morphological differences between organs collected either in dry or in rainy seasons. Each tubular cross-section in stage 1 was made of 0.5 type-A spermatogonia, 4.4 primary spermatocytes in preleptotene/leptotene, 3.7 in zygotene, 11.9 in pachytene, 35.6 round spermatids and 8.5 Sertoli cells. The mitotic and meiotic indexes were 15.4 and 2.9 cells, respectively, while the spermatogenesis yield was 68.7 cells. The testicular sperm reserves was 37.61×10^6^ cells, and daily sperm production per gram of testis averaged 209.68×10^6^ cells, both highest averages occurring in the rainy season. *S. lilium* male bats have a continuous reproductive pattern, high spermatogenesis yield and low support capacity by the Sertoli cells.

## Introduction

Bats show a diverse range of reproductive strategies, although their reproductive skills are directly related to individual body condition, which makes the availability of food a determining factor for the onset of the reproductive cycle in these animals [1], [2], [3].

The bat *Sturnira lilium* (E. Geoffroy, 1810) belongs to the Phyllostomidae family and presents essentially frugivorous eating habits, which make them great seed dispersers. The frugivory is highly beneficial for many plants, especially those from the Solanaceae family, which are of great ecological and economic importance. Additionally, some plant species depend on bat's behavior to their propagation [5], [6].

In general, bats inhabiting temperate or tropical regions show seasonal reproductive pattern, although some species may not be restricted to it [1], [3], [4]. In Brazil, *S. lilium* exhibits bimodal polyestry, with two breeding seasons per year: in October and March [4], [7]. These months correspond to the beginning and end, respectively, of the rainy season, which comprises the Spring and Summer [8]. In addition, some studies have related the reproductive activity of females, including pregnancy and lactation, to the rainy season [8]–[10].

The spermatogenic cycle dynamics allows us to infer more precisely about the reproductive activity of the species. The testicular weight is often related to the spermatic production [11], [12]. Additionally, the testicular size is intimate related to the reproductive behavior: monogamist or polygynous species (e.g. the majority of bats) shows smaller testes than promiscuous or polyandrous species [13], [14]. Besides, morphometrical and stereological analysis of the testicular parenchyma provide quantitative data of the spermatic production from the germ cell activity within the seminiferous epithelium [15].

Despite its wide distribution and ecological importance, *S. lilium* reproductive biology and spermatogenesis are poorly studied. Therefore, the aim of this study was to describe both the testicular dynamics in different seasons and the reproductive pattern of *S. lilium* using morphometric and stereologic tools.

## Materials and Methods

### Study area and animals collections

The sampling campaigns happened in Viçosa, located in Minas Gerais State (20°45′14″S and 42°52′55″W; 650 m altitude), from 2009 to 2010 (license number 214887-1/SISBIO/IBAMA). Viçosa is a mountainous city, inserted in the Atlantic Forest biome, classified as Cwa (Köppen) - mesotermic-humid, with rainy Summers and dry Winters. This regions shows low pluviosity from May to September and high pluviosity between December and March [16] and the 4 climatic seasons are not well defined.

Fourteen adult males *Sturnira lilium* were collected along 2009–2010 and put into two groups, representing the dry (Autumn and Winter; n = 7) and the rainy (Spring and Summer; n = 7) seasons. For the dry season 3 animals were collected during Autumn (June 2009; rainfall, temperature and air relative humidity ARH being 1.39 mm, 16.5°C and 83.56%, respectively) and 4 during the Winter (August 2009, rainfall, temperature and ARH being 0.44 mm, 17.55°C and 76.93%, respectively). For the rainy season 3 animals were collected during Spring (November 2009, rainfall, temperature and ARH being4.53 mm, 23.08°C and 75.43%, respectively) and 4 during Summer (January 2010, rainfall, temperature and ARH being 3.82 mm, 23.71°C and 73.12%, respectively) (Source: Meteorological Station, Department of Agricultural Engineering, Federal University of Viçosa).

The differentiation of the adult individuals was based on the fusion of the epiphysary cartilage of the fourth finger, in the metacarpophalangeal joint, following Kunz and Anthony methods [17]. The animals were kept into steel cages and received food (papaya and banana) and water *ad libitum*, from the day of capture to the day of euthanasia.

### Samples processing

The euthanasia was performed in the morning after the capture by using sodium pentobarbital injection (40 mg/kg), followed by injection of potassium chloride saturated solution. For the calculation of pentobarbital dosage, the cages were weighted separately and then with the animals, being discounted the animal' weight. After euthanasia, the animals were weighed again and the testes removed. The procedure described above was previously approved by the Ethics Commission of the Federal University of Viçosa (CEUA/UFV – number 93/011).

The testes were immersion fixed in Karnovsky solution [18] for 24 h and transferred to 70% ethanol. Both testes and the *tunica albuginea* of one of them were removed and weighed, being the *tunica albuginea* weight diminished from the gonad weight to calculate the volume of the testicular parenchyma. The gonadosomatic index (GSI) was calculated dividing the testes weight by the body weight, being the value multiplied by 100.

Testicular fragments were dehydrated in increasing ethanol series for inclusion either in glycol methacrylate (Historesin, Leica), for light microscopy analyses, or in Paraplast (Sigma), for detection of apoptotic cells. Semi serial sections (3 µm thick) were obtained using a rotary microtome (Leica RM2255), observing a minimum interval of 40 µm between different cuts. The preparations were stained with toluidine blue/sodium borate 1%. Morphometry and stereology were performed using digital images captured at different magnifications with the light microscope BX-40 Olympus. All images were analyzed by the software Image-Pro Plus.

For ultrastructural analysis, testicular fragments were fixed in Karnovsky solution for 1 hour and then transferred to a 2.5% glutaraldehyde solution for 24 h. After being rinsed with phosphate buffer, specimens were post-fixed in 1% osmium tetroxide in the same buffer for 2 h. Dehydration was performed in ethanol and acetone, followed by adding embedding resin (Epon 812). Ultrathin sections were contrasted with 3% uranyl acetate and 3% lead citrate and observed under a transmission electron microscope (JEOL 1011), in the Center for Microscopy and Microanalysis of Federal University of Viçosa (UFV).

### TUNEL and apoptosis counting

Apoptosis was detected by the TUNEL assay (Terminal deoxynucleotidyl transferase dUTP nick end labeling), which was performed according to the protocol of the detection kit (Calbiochem, Merck KGaA, Darmstadt, Germany).

Histological sections were deparaffinized, rehydrated and incubated with proteinase K for 20 min at room temperature, then washed in distilled water and incubated with hydrogen peroxide+methanol for 5 min, in order to stop endogenous peroxidase activity. The preparations were then incubated with Tdt equilibrium buffer in distilled water at room temperature and kept inside a humid chamber for 20 minutes. The incubation proceeded with Tdt mix enzyme and DTT for 60 min at 37°C. Immunoreactive cells were detected by incubating the slides with a mixture of 3,3-diaminobenzidino tetrachloride (DAB) and hydrogen peroxide for 13 min in a humid chamber protected from light. The sections were counterstained with hematoxylin, dehydrated in ethanol, cleared in xylene and mounted.

Preparations of the same material were used as negative and positive controls. TDT enzyme was omitted for the negative control, and apoptotic nuclei were stained with hematoxylin alone. The positive control slides were incubated with 1.00 U/µL DNAse I (Invitrogen) in DNAse buffer for 10 min before blocking endogenous peroxidase activity. Thus, the same steps were followed as described for the TUNEL technique. Preparations of different groups were stained at the same time in order to avoid discrepancies when comparing results.

The average number of apoptotic cells per tubular cross section was quantified considering cell counts per observed area (µm^2^) at 200× magnification. The identification of apoptotic cells was made based on nuclear characteristics (e.g. pyknosis) and DAB staining (brown color).

### Testicular morphometry

The volumetric ratio between the seminiferous tubules compartment and the interstitium was estimated by counting 3,520 points projected over 10 digital images (per animal, 100× magnification) obtained from histological slides. The volumetric proportions of all seminiferous tubule's components (*tunica propria*, seminiferous epithelium and lumen) and interstitium were calculated. The volume of the seminiferous tubules was estimated from the knowledge of its percentage within the testis and the knowledge of the volume of the testicular parenchyma. The volume of the seminiferous epithelium was obtained by multiplying its percentage within the seminiferous tubule by the tubular volume, dividing the result by 100.

The tubulesomatic index (TSI) was calculated in order to quantify the investment in the seminiferous tubules regarding to the total body mass. It was obtained by dividing the tubular volume by the body weight and multiplying the result by 100. The diameter of the seminiferous tubules and the height of the seminiferous epithelium were obtained from the measurement of 20 tubular cross-sections, regardless the stage of the cycle. The epithelium height was measured from the *tunica propria* to the tubular lumen.

The seminiferous tubules length (TL, in meters) per testis and per gram of testis was estimated using the formula: *TL = STV/лR^2^* (STV = seminiferous tubule volume; лR^2^ = area of the seminiferous tubules cross section; R = tubular radio). The TL was divided by the teste's weight in order to calculate the length of the seminiferous tubules per gram of testis.

### Germ cells counting

The population of each germ cell type was estimated by counting the germ cell nuclei and Sertoli cell nucleoli present in stage 1 of the seminiferous epithelium cycle (SEC) using 10 tubules cross-sections per animal. The nuclear diameters of 10 type-A spermatogonia (A) and 10 primary spermatocytes in zygotene (ZG) were measured, whereas 30 primary spermatocytes in preleptotene/leptotene (PL/L), primary spermatocytes in pachytene (PC), round spermatids (RS) and nucleoli of Sertoli cells (SC) were measured. Spermatogonia and zygotene spermatocytes are more scarce in the seminiferous epithelium, the reason why we only took 10 into account.

The cell population on stage 1 of the SEC was quantified based on the tubular morphology method [19]. The final averages were corrected considering the cut thickness along with the nuclear or nucleolar diameters of germ and Sertoli cells, respectively. The cells counting was taken according to the methodology proposed by Abercrombie [20], modified by Amann and Almquist [21].

The intrinsic efficiency of spermatogenesis was calculated from the rates found between the corrected numbers of germ cells, in order to quantify the efficiency of spermatogenesis. Since PL/L and ZG are a subpopulation of initial primary spermatocytes (IPS), these two cell types were pooled for calculation of mitotic index, or the also known coefficient of spermatogonial mitosis (IPS/A). This coefficient is important in order to quantify the loss or degeneration occurring during the spermatogonial phase. The meiotic (RS/PC), which determines the efficiency of meiotic divisions, and the spermatogenesis yield (RS/A), which quantifies the efficiency of the spermatogenic process as a whole, were also calculated. The Sertoli cells index (SCI), which indicates the carrying capacity of this cell type by the total number of germ cells was determined from the rates found between the corrected number of germ cells and the corrected number of Sertoli cells, obtained in stage 1 of the SEC ((A+PL/L+ZG+PC+RS)/SC).

Assuming that the cell loss is not significant in spermatogenesis, the testicular sperm reserves (TSR) was calculated based on the round spermatids population in stage 1 of the SEC. Thus, the average number of round spermatids was taken in seminiferous tubules cross sections of known thickness. This number was corrected for the total length of the tubule per testis or per gram of testis, using the following formula: *TSR = (length of the seminiferous tubules/cut thickness)×corrected number of round spermatids per cross section* [19].

The daily sperm production (DSP) was calculated by dividing the TSR by the seminiferous epithelium cycle duration, which is 15.52 days [44]. The DSP per gram of testis was obtained by dividing the previous value by the testes' weight.

### Statistical analysis

The values were tested for normality using the Lilliefors test, whereas the Cochran test was used to test homogeneity. The data that met the parametric assumption were compared using the Student t test. On the other hand, the values that were not normally distributed were analyzed with the Wilcoxon non-parametric test. Statistical analyses were made using the software STATISTICA. All results were grouped as mean ± standard error of the mean (p<0.05).

## Results

### Morphometry and stereology

The biometric results as well as the volumetric proportions of the components of the testicular parenchyma of *Sturnira lilium*, in wet and dry seasons, are listed in Table 1. The average body weight observed in both dry and wet seasons was 23 g, whereas the testicular weight was 0.06 g, thus resulting in a GSI of 0.27%. The *tunica albuginea* represented 10% of testicular weight, while the testicular parenchyma was composed of 85% of the seminiferous tubules and 15% of interstitium. Comparing the dry and rainy seasons, none of the parameters analyzed for testicular morphometry showed significant changes. The *tunica propria* represented 3.7% of the tubular compartment, followed by 69.1% of the seminiferous epithelium and 12.1% of the lumen.

**Table 1:** Biometry, morphometry and stereology of the testicular components of *Sturnina lilium*, during dry and rainy seasons.

Parameters	Dry season	Rainy season	Mean ± SE
Body weight (g)	22.58±0.46	24.08±0.73	23.33±0.53
Testes weight (g)	0.05±0.01	0.08±0.02	0.06±0.01
Gonadosomatic index (%)	0.23±0.03	0.32±0.08	0.27±0.03
*Tunica albuginea* (%)	8.82±1.51	12.05±0.67	10.43±1.14
Seminifeours tubules (%)	85.38±2.06	84.64±3.68	85.01±0.26
*Tunica propria* (%)	3.70±0.48	3.79±0.15	3.74±0.03
Epithelium (%)	71.44±2.01	66.82±2.33	69.13±1.63
Epithelium volume (mL)	0.03±0.005	0.04±0.013	0.04±0.005
Lumen (%)	10.24±1.00	14.02±2.24	12.13±1.34
Interstitium (%)	14.62±2.06	15.37±3.68	14.99±0.27
Seminiferous tubules (mL)	0.04±0.01	0.06±0.02	0.05±0.01
Interstitium (mL)	0.006±0.001	0.007±0.0004	0.007±0.0002
Seminiferous tubule diameter (µm)	134.42±7.89	144.26±9.57	139.54±3.62
Seminiferous epithelium height (µm)	39.41±1.91	40.96±2.89	40.19±0.55
Tubulesomatic index (%)	0.18±0.03	0.25±0.07	0.21±0.03
Length of seminiferous tubules per testis (m)	3.06±0.36	3.98±0.46	3.52±0.33
Length of seminiferous tubules per gram of testis (m/g)	76.80±14.73	82.47±21.29	79.63±2.22

Lines with different superscripts indicate statistical difference between values. Data are expressed by Mean ± standard error of the mean (SE).

The volume of the germ epithelium was 0.04 mL, whereas the volume of the seminiferous tubules was 0.05 mL, thus resulting in a 0.21% TSI. The tubular diameter and the height of the seminiferous epithelium were 139.3 and 40.1 µm, respectively. The animals had 3.5 m of seminiferous tubules per testis and 79.6 m of seminiferous tubules per gram of testis.

**Figure 1:**
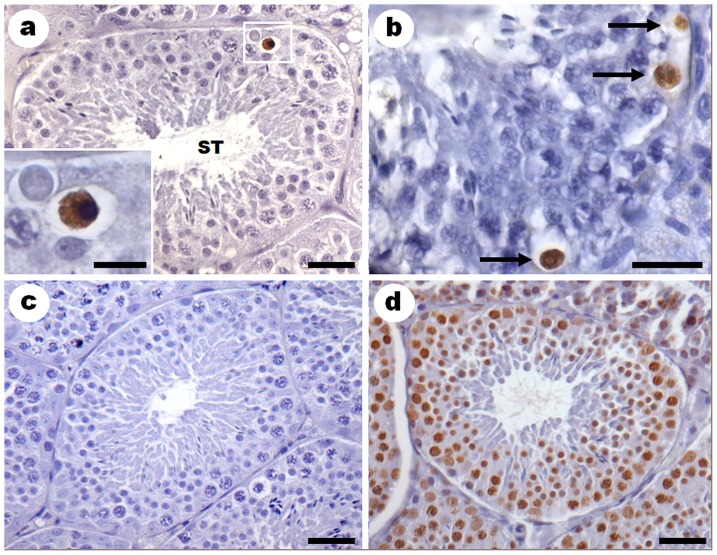
*Sturnira lilium* testis submitted to TUNEL technique. Brown nuclei are positive to the TUNEL assay. **a**) Dry season. **ST**: Seminiferous tubule. Bar: 30 µm. Inset: pachytene spermatocyte going throw apoptosis. Bar: 10 µm. **b**) Rainy season. Arrow: Pachytene spermatocytes undergoing apoptosis. Bar: 20 µm. **c**) Negative control. Bar: 30 µm. **d**) Positive control. Bar: 30 µm.

There was no significant variation on the number of apoptotic cells between seasons: 0.63 and 1.66 apoptotic cells per µm^2^ in dry and rainy seasons, respectively. Spermatocytes in pachytene were the most frequently apoptotic cell type (Fig. 1).

### Cell counting

**Figure 2:**
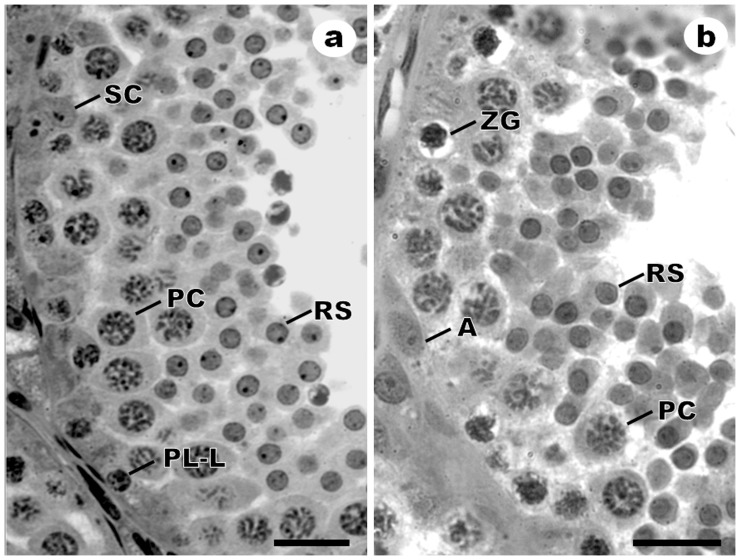
Light microscopy of seminiferous tubules cross-sections in the beginning (a) and in the end (b) of stage 1 of seminiferous epithelium cycle in *Sturnira lilium*. **a**) Dry season. **PL/L**: Preleptotene/Leptotene spermatocytes; **PC**: Pachytene spermatocyte; **RS**: Round spermatid; **SC**: Sertoli cell's nucleus. Bar: 30 µm. **b**) Rainy season. **A**: Type-A spermatogonia; **ZG**: Zygotene spermatocyte; **PC**: Pachytene spermatocyte; **RS**: Round spermatid. Bar: 20 µm.

Figure 2 shows seminiferous tubules cross sections with typical cellular arrangement within the seminiferous epithelium at stage 1 of the SEC. Initial primary spermatocytes are present both in preleptotene/leptotene at the beginning of stage 1 (Fig. 2a) and in zygotene at the end of stage 1 (Fig. 2b), as well as of other cell types that make up the germinal epithelium at this stage.

The corrected number of each cell type in stage 1 are shown in Table 2. On average, a total of 8.5 Sertoli cells, 0.5 type-A spermatogonia, 4.4 preleptotene/leptotene spermatocytes, 3.7 zygotene spermatocytes, 11.9 pachytene spermatocytes and 35.6 round spermatids were recorded. Regarding the initial population of primary spermatocytes, the seminiferous tubules that were at the start of stage 1 contained only preleptotene/leptotene spermatocytes, while those at the end of stage 1 already showed zygotene primary spermatocytes (Fig. 2). The simultaneous occurrence of these two cell types was not detected so often, although when it happened the cells occupied opposite portions of the seminiferous tubule. The number of cells in zygotene was significantly higher during the rainy season (p = 0.03), although it did not influence the spermatogenic yield (Table 2).

**Table 2:** Corrected germ cell populations in stage 1 of the seminiferous epithelium cycle and sperm production indexes of the bat *Sturnira lilium*, during dry and rainy seasons.

Parameters	Dry season	Rainy season	Mean ± SE
Sertoli cell	8.15±0.45	8.88±0.67	8.51±0.36
Type-A spermatogonia	0.48±0.05	0.66±0.21	0.57±0.09
Initial primary spermatocyte	7.24±0.90	9.19±1.56	8.21±0.98
Preleptotene/leptotene	4.58±0.82	4.37±0.94	4.47±0.10
Zygotene	2.66±0.77^a^	4.82±0.99^b^	3.74±1.08^a^
Late primary spermatocyte: pachytene	11.00±0.83	12.96±2.29	11.98±0.98
Round spermatid	34.33±5.05	37.05±7.09	35.69±1.36
Mitotic index	16.10±2.67	14.85±3.71	15.48±0.62
Meiotic index	3.05±0.25	2.82±0.19	2.94±0.11
Spermatogenesis yield	75.59±13.01	61.92±11.18	68.76±6.84
Sertoli cell index	6.74±1.06	7.39±1.77	7.06±0.33
Sertoli cell per testis (×10^12^)	8.39±1.19	12.14±1.95	10.26±1.87
Sertoli cell per gram of testis (×10^13^)	18.48±3.79	26.14±7.94	22.31±3.82
Daily sperm production (×10^6^)	9.20±0.46^a^	12.60±0.88^b^	10.90±1.69^a^
Daily sperm production per gram of testis (×10^6^)	193.50±22.95	225.87±43.16	209.68±16.18
Testicular sperm reserves (×10^6^)	31.75±1.58^a^	43.47±3.05^b^	37.61±5.86^a^
Testicular sperm reserves per gram of testis (×10^7^)	75.12±9.09	77.92±14.98	76.52±1.39

Lines with different superscripts indicate statistical difference between values. Data are expressed by Mean ± standard error of the mean (SE).

Sertoli cells exhibited nucleolar average diameter of 1.9 µm. Type-A spermatogonia, primary spermatocytes in preleptotene/leptotene, in zygotene, in pachytene and round spermatids showed mean nuclear diameter of 9.7, 6.6, 7.4, 9.0 and 5.1 µm, respectively.

### Ultrastructure of germ cells

**Figure 3:**
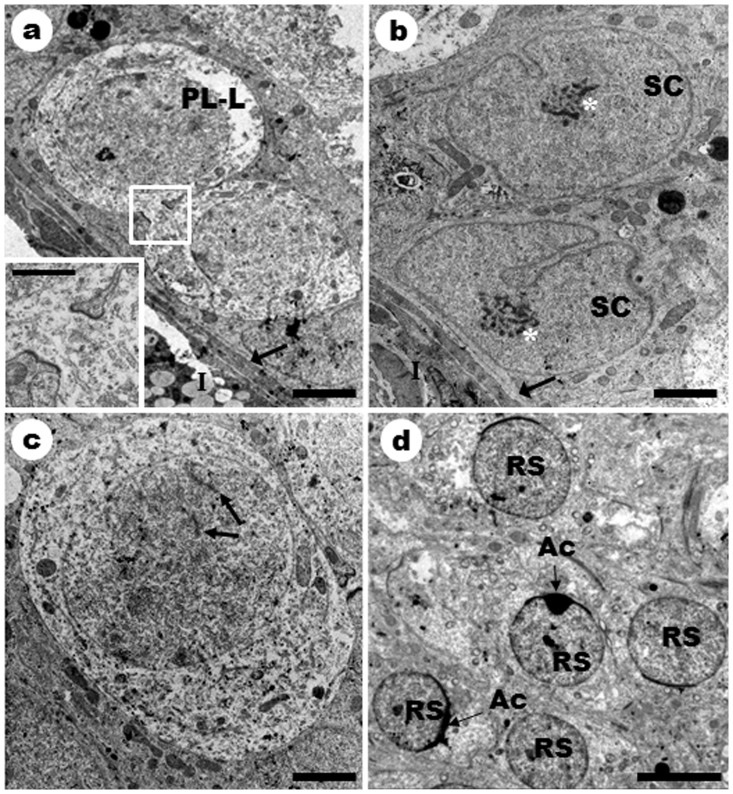
Ultrastructure of the germ cells in stage 1 of the seminiferous epithelium cycle in *Sturnira lilium*. **a**) Dry season. **PL/L**: Preleptotene/Leptotene spermatocytes connected by cytoplasmic bridge; **→**: Basal lamina; **I**: Interstitium. Inset: Cytoplasmic bridge. Bars: 3 µm. **b**) Dry season. **SC**: Sertoli cell's nucleus; _*_: Sertoli cell's nucleolus; **→**: Basal lamina; **I**: Interstitium. Bar: 2 µm. **c**) Rainy season. **→**: Linear chromatin regions inside the nucleus of a leptotene primary spermatocyte. Bar: 2 µm. **d**) Rainy season. **RS**: Round spermatid nucleus; **Ac**: Acrosome. Bar: 5 µm.

The ultrastructure of all cell types present within the germinal epithelium on stage 1 is shown in Figure 3. Preleptotene/leptotene primary spermatocytes connected by cytoplasmic bridges (Fig. 3a) and Sertoli cells with characteristic nuclei and fragmented nucleoli were evident (Fig. 3b). These cell types were located near to the basal lamina. Primary spermatocytes' nuclei in leptotene showed linear chromatin regions, which were mismatched elements that form the synaptonemal complex. At this phase, the spermatocyte is no longer in contact with the basal lamina (Fig. 3c). Round spermatids were also observed showing the acrosomal cap covering part of the nuclear surface (Fig. 3d).

### Sperm production indexes

Sperm production indexes are listed on Table 2. The mitotic index revealed that 15.4 primary spermatocytes were produced from a type-A spermatogonia. Meiosis yield was 2.9 spermatids per primary spermatocyte, while the general spermatogenesis yield was 68.7 cells, considering both dry and rainy seasons. These parameters did not change significantly between dry and rainy seasons.The testis of *S. lilium* showed an average of 10.26×10^12^ Sertoli cells per testis, while in each gram of testis an average of 22.31×10^13^ Sertoli cells could be found. Considering all germ cells, a single Sertoli cell in *S. lilium* is able to support 7 germ cells.

### Testicular sperm reserves and daily sperm production

The sperm reserve found in each testis was significantly higher in the rainy season (p = 0.0259), while the sperm reserves per gram of testis did not vary significantly between groups, and averaged 76.52×10^7^ cells (Table 2). The daily sperm production of *S. lilium* was 10.90×10^6^ sperm, being significantly higher (p = 0.0156) in the rainy season, although showing no significant differences per gram of testis (Table 2).

## Discussion

### Morphometry and stereology

The present study provides valuable data for better understanding of the reproductive biology of the order Chiroptera, with emphasis on morphometry and stereology apllied to spermatogenesis, since only one previous study regarding the theme has been published so far [22]. Therefore, we often use other mammalian groups results to better disscuss our findings.

The GSI found for *Sturnira lilium* corroborates the idea of polygyny, since it was approximately 1.6 times lower than the observed in other polygynous species, such as *Molossus molossus* and *Artibeus lituratus* [22], [23], although 5.4 times higher than that observed in animals essentially monogamous as the crab-eating fox *Cerdocyon thous* [24], or the optional monogamic maned wolf *Chrysocyon brachyurus* [25].

Similarly, the body weight observed in animals in this study was similar to that previously reported for the same species in Brazil (21 g) [26].

The testicular histoarchitecture of *S. lilium* followed the organization pattern observed in other bats [22], [27]–[30], and mammalian species [12], [15], [24], [25], [31]–[35]. The percentage of testicular parenchyma represented by the *tunica albuginea* was in agreement with that reported for several mammalian species [12].

The seminiferous tubules compartment of *S. lilium* occupied 5% less area than in *M. molossus*, while the interstitial area was about 40% higher [22]. Both values are within the range reported for several mammals, where the percentage of seminiferous tubules varies 60–90%, and the percentage of interstitium 11–40% [12].

The tubular diameter values were close to the lowest values observed for other mammals, whose range is 133–300 µm [12], [22], [23], [31], [32], [35]–[37]. Similarly, the height of the seminiferous epithelium was among the lowest ever observed values for mammals, from 31 to 100 µm [22]–[24], [31], [34], [37]–[39]. Thus, after combining these values, we observed a high tubular length per gram of testis, which in *S. lilium* was about 5 times higher than the commonly in most mammals: 15 m [12], [15], [33], [36]. High tubular length per gram of testis was also found in bat *M. molossus* (48 m) [22] and *A. lituratus* (27 m) [23], indicating that this is a common feature among bats. The highest values previously reported for tubular length per gram of testis were found in the agouti *Dasyprocta leporina* and in paca *Agouti paca*, with 32 and 35 m, respectively [39].

Along with the tubular length, the TSI was 5 times higher than the observed to larger mammals species, such as *C. thous* [24] and the oncilla *Leopardus tigrinus* [35]. However, TSI in *S. lilium* was about 1.8 times lower than that reported for other Chiroptera [22] and 2.4 times lower than that reported for the mouse [37]. The high tubular length and TSI reinforce the trend described by Kenagy and Trombulak [13] that small animals have greater investment in sperm production. Since most of the parameters analyzed for testicular morphometry showed no significant changes between the dry and rainy seasons, we suggest that *S. lilium* presents a continuous annual reproductive pattern. This reproduction pattern may be related to the wide availability of food that can be found throughout the year [6], [40], [41]. All animals used in this study showed full-developed germinal epithelium and all stages of the SEC were present. Tubular length per gram of testis and TSI levels may also be related to this continuous reproductive pattern, which requires greater investment in the seminiferous tubules to support spermatogenesis throughout the year.

### Germ cell counting

The germ cell types commonly found in stage 1 of the SEC are: type-A spermatogonia, primary spermatocytes in preleptotene/leptotene, primary spermatocytes in pachytene and round spermatids, all of them being supported by the Sertoli cells [12], [29], [35], [38], [39], [42], [43]. However, *S. lilium* germ epithelium also contained primary spermatocytes in zygotene, what was similar to the findings described in *M. molossus* [22]. This fact occurs due to the longer duration of this stage, as described earlier to the same species [44].

Cell counts were very similar to that found in *M. molossus* [22]. A low number of type-A spermatogonia was recorded (0.48) when compared to other wild species such as in the deer *Mazama gouazoubira* (1.1) [34] and the peccary *Tayassu pecari* (2.4) [45]. The counting of the remaining germ cells that comprise the seminiferous epithelium at stage 1 was also below the observed for other mammalian orders, such as the jaguar *Panthera onca* [46]. On the other hand, the number of Sertoli cells by tubular cross section was high, being the average of 8.5 cells was found for *S. lilium* above that of animals like *M. gouazoubira* (3.7) [34] and *C. brachyurus* (7.7) [43], and close to that found in dogs (8.1) [47].

### Ultrastructure of germ cells

The presence of cytoplasmic bridges between primary spermatocytes at preleptotene/leptotene illustrates the fact that all germ cells show this type of connection, which allows a synchronous cellular development. Thus, the various cell generations observed in the seminiferous epithelium along the tubular cross-section are in a sequence of increasing maturation, initiating from the base to the tubular lumen [48].

The nuclei of the Sertoli cells were irregular in shape with typical indentations, corroborating previous descriptions in other bats [49], [50], and mammals species [48], [51]. The nucleoli of Sertoli cells showed themselves granular, similar to that observed in the bat *Desmodus rotundus* [49].

The linear regions of chromatin observed in primary leptotene spermatocytes are, according to Russell et al. [48], unpaired elements that form the synaptonemal complex from the primary spermatocyte in zygotene. These are complex protein structures that connect pairs of homologous chromosomes and are essential for the progression of the first meiotic division [52], [53]. The features disclosed in the primary spermatocyte leptotene were similar to those described by Beguelini et al. [50] for the bat *Platyrrhinus lineatus* and by Russell et al. [48].

The presence of round spermatids is a hallmark of stage 1 of the SEC. Despite the light microscopy did not allow us to clearly observe the presence of the acrosome, the ultrastructure showed that at this stage of the cycle a acrosomal cap exists and covers part of the nuclear surface of the spermatid, similar to that described in *M. molossus* [54].

### Sperm production indexes

There are few studies about the number of spermatogonial generations present in bats. Five spermatogonia generations were described to the megachiropteran *Rousettus leschenaulti*, while studies in pets reveal the existence of six generations [55]. Thus, the division of the first generation of spermatogonial stem cells originate, at the end of the process of mitotic divisions, 64 primary spermatocytes [12], [48]. In *S. lilium*, 15.4 initial primary spermatocytes were produced from each type-A spermatogonia and the estimated cell loss of 76% was observed in the mitotic phase. This value is within the range of 60 to 90% observed for most domestic and wild animals [12], [34], [45], [46], [56]. However, the lowest cell loss in most mammals occurs during the meiotic phase [12], [57]. The losses are related to apoptosis in spermatocytes, as part of the mechanism for maintaining an appropriate number of germ cells and elimination of genetically abnormal cells [58], [59]. In general, this loss in mammals is about 25%, or from every four round spermatids expected, three are formed [12]. In *S. lilium* 26% of the cells were lost, and the meiotic yield was within the range previously reported for other mammals (1.90 to 3.62 cells) [12], [34], [37], [43], [45], [46], [56], [60]. Counting of apoptotic cells revealed no significant differences between the seasons.

As losses during spermiogenesis are not considered significant, the calculation of spermatogenesis efficiency, because it is based on the population of round spermatids, provides high reliability for the assessment of sperm production [11], [12], [19], [61], [62]. Thus, the quantitated number of round spermatids in stage 1 is considered as the final population of sperm [47]. In *S. lilium*, about 68 round spermatids were produced per each spermatogonia, which is related to higher sperm reserves found in this bat species. This value is above the decribed for many domestic and wild mammals [12], [34], [45], [46].

The Sertoli cell's index found for *S. lilium* was 1 to 3 times lower than reported for most mammalians, with values ranging from 10 to 22 germ cells for each Sertoli cell [12], [34], [45], [63], [64]. This finding indicates low support capacity by these cells in *S. lilium*, as observed in *M. molossus* [22]. Also, the number of Sertoli cells per gram of testis was considerably higher (8.9 times) than the average 25×10^6^ cells found in other mammals [12], [34], [37]. This data shows that a large number of cells is needed to sustain spermatogenesis, which explains the lower support capacity observed in *S. lilium*.

### Testicular sperm reserves and daily sperm production

It is known that the bats, as well as other mammals, adjust its reproductive cycle in accordance with the environmental conditions [7]. *S. lilium* offspring is born during the rainy season, which includes Spring and Summer in the Southern hemisphere, therefore the pups have access to more favourable temperatures and food resources [8], [10], [65]. As *S. lilium* pregnancy period lasts four months [66], [67], it is consistent to assume that matings occur more frequently during the dry season, so that births happen on the next season. Therefore, TSR and DSP increasing during the rainy season in *S. lilium* may indicate the occurrence of seminiferous epithelium recovery after the mating period.

In *S. lilium*, DSP per gram of testis was 4–12 times higher than those found in animals such as agouti (52×10^6^) [39], the paca (39×10^6^) [39] and the jaguar (17×10^6^ to 27×10^6^) [33], [60], corroborating the Kenagy and Trombulak statement [13]: the smaller the animal, the higher the sperm production.

Considering our findings, we can conclude that *S. lilium* shows a continuous reproductive pattern along the year in Southeastern Brazil. The species shows one of the highest seminiferous tubules lengths and daily sperm production recorded for mammals. The fact that each Sertoli cell have supported a low number of germ cells can characterize a strategy to minimize cell loss, since spermatogenesis yield was high.
